# From Structure to Catalysis: Recent Developments in the Biotechnological Applications of Lipases

**DOI:** 10.1155/2014/684506

**Published:** 2014-03-24

**Authors:** Cristiane D. Anobom, Anderson S. Pinheiro, Rafael A. De-Andrade, Erika C. G. Aguieiras, Guilherme C. Andrade, Marcelo V. Moura, Rodrigo V. Almeida, Denise M. Freire

**Affiliations:** Departamento de Bioquímica, Universidade Federal do Rio de Janeiro, Avenida Athos da Silveira Ramos, 21941-909 Rio de Janeiro, RJ, Brazil

## Abstract

Microbial lipases are highly appreciated as biocatalysts due to their peculiar characteristics such as the ability to utilize a wide range of substrates, high activity and stability in organic solvents, and regio- and/or enantioselectivity. These enzymes are currently being applied in a variety of biotechnological processes, including detergent preparation, cosmetics and paper production, food processing, biodiesel and biopolymer synthesis, and the biocatalytic resolution of pharmaceutical derivatives, esters, and amino acids. However, in certain segments of industry, the use of lipases is still limited by their high cost. Thus, there is a great interest in obtaining low-cost, highly active, and stable lipases that can be applied in several different industrial branches. Currently, the design of specific enzymes for each type of process has been used as an important tool to address the limitations of natural enzymes. Nowadays, it is possible to “order” a “customized” enzyme that has ideal properties for the development of the desired bioprocess. This review aims to compile recent advances in the biotechnological application of lipases focusing on various methods of enzyme improvement, such as protein engineering (directed evolution and rational design), as well as the use of structural data for rational modification of lipases in order to create higher active and selective biocatalysts.

## 1. Introduction

Lipases (triacylglycerol acyl hydrolases, E.C. 3.1.1.3) are enzymes that catalyze the hydrolysis of fats and oils with release of free fatty acids, diglycerides, monoglycerides, and glycerol. Furthermore, in organic media, these enzymes also catalyze synthetic reactions including esterification, acidolysis, alcoholysis, and interesterifications [1, 2]. Lipases may act under mild conditions, are highly stable in organic solvents, show broad substrate specificity, and usually show high regio- and/or stereoselectivity in catalysis. This versatility makes lipases one of the most widely used group of biocatalysts for biotechnological processes [3–5]. Lipases find applications in food modification, detergent formulation, cosmetic, pharmaceutical, leather, textile, and paper industries, biodiesel and biopolymer production, or pretreatment of lipid-rich wastewaters [1, 6]. These enzymes are found in many species of animals, plants, bacteria, and fungi. Moreover, microbial lipases are the most appealing ones mainly because of their applied properties, such as versatility and ease of mass production [5, 6].

## 2. Biotechnological Applications of Lipases

### 2.1. Hydrolysis of Oils and Fats: Detergents, Pulp and Paste, and Leather

The most commercially important field for lipase application is their incorporation to detergents, which are used mainly in industrial laundry and household dishwashers to remove fat-containing stains [3]. The detergent lipases mostly used are the ones from* Thermomyces* sp., expressed in recombinant strains of* Aspergillus oryzae* (Lipolase, Novozymes), as well as from* Pseudomonas *spp. (Lumafast and Lipomax, Gist-Brocades). The requirements for lipase application in detergent industries may include high broad substrate specificity, activity and stability at alkaline pH and temperatures above 40°C, and compatibility with different components in a detergent, including metal ions, surfactants, oxidants, and proteases [5, 7, 8]. Another application of these enzymes is in the removal of “pitch” (hydrophobic components of wood, namely, triglycerides and waxes) from pulp produced in the paper industry. This enzymatic pitch control method has been used in large scale since early 1990s [3, 5]. Lipases can also be used to remove fats and grease from skins and hides. This process generates a higher-quality product (more uniform colour and a cleaner appearance) when compared to leather that is manufactured by traditional methods [5].

### 2.2. Pretreatment of Lipid-Rich Wastewaters

Wastewaters from dairies, slaughterhouses, and fish-processing contain high levels of fats and proteins with low biodegradability that may cause serious environmental damage if not properly treated [9, 10]. An enzymatic hydrolytic step before further biological treatment can reduce the particles diameter, increasing their surface area and favoring organic matter assimilation by the microbial consortium [11]. Thus, the application of lipases in wastewater treatment may improve biological degradation of fatty wastewaters, thereby accelerating this process [10]. Nevertheless, this added pretreatment procedure becomes economically unfeasible if there are high costs associated with the commercial enzymatic preparations. Therefore, the development of low-cost enzyme preparations becomes essential.

### 2.3. Food Processing and Improving Quality

In dairy industry, microbial lipases have been used in selective hydrolysis of fat triglycerides to release free fatty acids, which are used to develop flavored products, such as cheese, butter, margarine, milk chocolate, and sweets [3]. Certain microbial lipases are highly regio- and fatty acid-specific and thus are commonly used for the production of new oils and fats through interesterification reactions [1]. These reactions are used to produce high added value products, such as cocoa butter equivalents, human milk fat substitutes, pharmaceutically important polyunsaturated fatty acids (PUFAs), rich/low calorie lipids, and “designers fats” or “structured lipids.” Enzymatic transesterification can also be used to modify the properties of triacylglycerol mixtures and produce fats with optimum melting characteristics and free of trans fatty acids, targeting commercial applications for food industries. Sellami et al. investigated the production of a trans-free fat, for margarine formulation, by enzymatic transesterification of palm stearin and palm olein blends. The margarine prepared with transesterified fat showed similar spreadability to that of a commercial product [12]. The major attractions for lipase utilization are energy saving and the decrease of lipid degradation due to high temperatures [5, 13].

### 2.4. Synthesis of Fine Chemicals (Therapeutics, Agrochemicals, Flavors and Fragrances, Emulsifiers, and Cosmetics)

Stereospecificity is especially important for the synthesis of bioactive molecules, as chirality is a key factor in the efficacy of many drugs and usually only one of the enantiomeric forms manifests bioactivity. In this context, the use of lipases to synthesize the chiral building blocks for the production of pharmaceuticals, agrochemicals, and pesticides with high enantiomeric purity has become widespread. These biocatalysts offer several advantages over traditional chemical routes, such as practicality, high efficiency, selectivity, easy separation from the unreacted substrates, and mild reaction conditions [5, 14–16]. It is important to highlight that lipase cost does not restrict the production of added value products, such as pharmaceuticals. The kinetic resolution of (R,S)-1,2-isopropylidene glycerol ester derivatives (solketal) catalyzed by different lipases was investigated by Machado et al. [14]. Solketal is an important chiral synthon in the synthesis of diglycerides, glyceryl phosphates, tetraoxaspiroundecanes, and many biologically active compounds, such as glycerolphospholipids, *β*-blockers, prostaglandins, and leucotrienes.* Pseudomonas* sp. lipase (Amano AK) was proven to be the most effective in promoting the resolution of (R,S)-IPG esters yielding 22% of (R)-IPG octanoate (99% ee) in 48 h at 30°C. Cunha et al. studied the enzymatic kinetic resolution of (±)-1,2-O-Isopropylidene-3,6-di-O-benzyl-myo-inositol (precursor of chiral myo-inositol derivatives (inositol phosphates and their analogs)) by Novozym 435. The use of vinyl acetate as acylating agent has increased the yield to over 49%, while maintaining a very high ee (>99%) [17]. Similar results were obtained by Manoel et al. [18] in the kinetic resolution of DL-1,3,6-tri-O-benzyl-myo-inositol by* Pseudomonas* spp. lipases (PS-C, PS-IM) and Novozym 435, where the O-acylated L enantiomorph was obtained in up to >99% ee with conversions up to >49% [18]. In another work, Manoel et al. investigated the biocatalytic continuous flow process in a packed-bed reactor (PBR) for the kinetic resolution of (±)-1,3,6-tri-O-benzyl-myo-inositol ((±)-1) by alcoholysis using Novozym 435 [15]. Excellent conversions and enantiomeric ratios (50% conversion and eep >99%) were attained in short reaction time (3 min of residence time) using TBME (*tert*-butyl-methyl-ether) as solvent and vinyl acetate as acetylating agent. The lipase remained stable over a longer period of time (9-cycle experiment). Lipases are currently being used by many pharmaceutical companies world-wide for the preparation of optically active intermediates on a kilogram scale [5]. Despite the large number of publications in this field, the number of industrial enantioselective processes based on lipase catalysis is still limited.

Organic esters from short-chain fatty acids are amongst the most important natural fragrances and flavors. Examples of these esters include anthranilic acid alkyl esters (which possess sweet and orange odor with a pungent taste), butyl butyrate, isoamyl acetate (banana flavor), ethyl valerate (green apple flavor), and butyl acetate (pineapple flavor) [19]. The use of lipases leads to better quality products suitable to fragrance and flavor industry [20].

Monoacylglycerols (MAGs) are nonionic surfactants and constitute the main category of emulsifiers used in pharmaceutical and cosmetic formulations as well as prepared foods [19]. Lipases can be used to overcome the drawbacks (dark color, thermal degradation, and burnt taste) of the chemical glycerolysis that is carried out at high temperatures (220–250°C) employing alkaline catalysts and high pressures [6]. The enzymatic synthesis of MAGs can be performed by selective hydrolysis using 1,3-regiospecific lipases, glycerolysis of fats or oils, and esterification of fatty esters with glycerol. da Silva et al. reported the synthesis of monocaprin by direct esterification of glycerol with capric acid catalyzed by a commercial immobilized lipase (Lipozyme IM 20) [21]. The composition of the final product met the requirements established by the World Health Organization food emulsifiers (61.3% monocaprin, 19.9% dicaprin, and 18.8% capric acid).

Tyrosyl lipophilic derivatives are antioxidants with application in food, cosmetic, and pharmaceutical industries. Aissa et al. studied the synthesis of tyrosyl fatty acid esters by direct esterification of tyrosol with different fatty acids (from C2 to C18:1) using Novozyme 435 as biocatalyst. Tyrosyl esters derivatives TyC8, TyC10, and TyC12 exhibited antibacterial and antileishmanial activities [22].

### 2.5. Production of Biofuels and Biolubricants

Biodiesel has attracted considerable interest in recent years as an alternative and renewable energy source. Nowadays, particular attention has been drawn to the use of lipases as biocatalysts for biodiesel synthesis. These enzymes may substitute conventional alkaline processes that generate undesirable byproducts, render the separation of the catalyst from glycerol difficult, produce highly alkaline waste, and use high-quality raw materials [23, 24]. Lipases can catalyze both the transesterification of triacylglycerols and the esterification of free fatty acids to yield monoalkyl-esters. They perform their catalytic activity with oils from different origins, including waste oils with a high acidity and water content. Furthermore, the enzymatic process enables easy separation from the byproduct, glycerol [25, 26]. The enzymatic biodiesel production has been carried out by several research groups using both extracellular and intracellular lipases as well as raw materials from different sources (animal fat, vegetable oil, used cooking oil, and acid waste from vegetable oils production) [24, 27–30]. These reports show good results with conversion ranges above 90%. However, enzyme's high cost remains a barrier for enzymatic production of biodiesel [23]. Efforts have been focused on the development of low-cost enzyme preparations and more stable and active biocatalysts, in order to improve conversion yields in a shorter period of time and to recycle the enzyme for as much batches as possible [13].

Biolubricants are another group of great industrial interest in which lipases can be used as biocatalysts. The patent EP 2657324 A1 [31] describes an enzymatic process for production of biolubricant from methyl ricinoleate (biodiesel from castor oil) and/or from a mixture of methyl oleate and linoleate (biodiesel from* Jatropha* oil) by transesterification with trimethylolpropane. Conversions of 80% to 99% of the ester (castor oil and* Jatropha* oil biodiesel) were achieved and the product showed good properties of viscosity, viscosity index (VI), pour point, and oxidation stability. Aguieiras et al. have investigated biolubricant synthesis from oleic acid and methyl ricinoleate using immobilized commercial lipases [32]. Novozym 435 showed the best performance and the synthesized product exhibited good values of pour point, viscosity, and viscosity index.

### 2.6. Production of Biodegradable Polymers

Another field for lipases application is the synthesis of useful and biodegradable biopolymers like polyesters, produced from renewable natural resources [33]. Lipase-catalyzed polymerization reactions are classified into two major polymerization modes: ring-opening polymerization [34] and polycondensation. The latter may include polycondensation of dicarboxylic acids or their derivatives with diols and polycondensation of oxyacids or their esters [33]. This polymerization approach has been successfully employed by several works through a range of strategies.* Rhizomucor miehei* lipase was used in the polymerization of bis(2,2,2-trifluoroethyl) sebacate and aliphatic diols [33, 34]. Mahapatro et al. have studied the polymerization of 12-hydroxydodecanoic acid catalyzed by Novozym 435 and obtained conversion of 91% in oligomers of high molecular weight [35]. Polyesters of ricinoleic acid with 72% of conversion were synthesized by Bódalo et al. using immobilized lipase from* Candida rugosa *[36]. These results indicate the applicability of lipases for polymerization reactions through different pathways.

Taking into account the wide range of applications and the importance of lipases in biotechnology, many efforts have been directed toward the understanding of how these enzymes work at the molecular and atomic level. Since the BRIDGE-T lipase project (1990–1994) until today, numerous three-dimensional structures of lipases from several different organisms have been reported, shedding light onto the mechanism used by these enzymes during catalysis.

## 3. Structural Features of Lipases

Despite their low primary sequence identity, lipases display a very similar fold. Other enzymes, such as esterases, proteases, dehalogenases, epoxide hydrolases, and peroxidases, exhibit similar structural features and, altogether, they constitute the *αβ* hydrolase family [37]. The *αβ* hydrolase fold is characterized by the presence of a central *β*-sheet containing eight parallel *β*-strands, with the exception of *β*2, which is antiparallel with respect to the others. The central *β*-sheet has a left-handed superhelical twist, generating a 90° angle between the first and the last strands. Strands *β*3 to *β*8 are connected by a bundle of helices: helices A and F pack against the concave side of the central *β*-sheet, while helices B, C, D, and E pack against the convex side [38, 39]. The active site of *αβ* hydrolases consists of a highly conserved catalytic triad: one nucleophilic residue (serine, cysteine, or aspartic acid), one catalytic acidic residue (aspartic or glutamic acid), and one histidine residue. In lipases, the nucleophile has always been characterized as a serine residue [39–41].

### 3.1. Active Site

The nucleophilic residue is located in a highly conserved pentapeptide Sm-X-Nu-X-Sm, where Sm—small residue, usually a glycine, which may occasionally be substituted by alanine, valine, serine, or threonine; X—any residue; Nu—nucleophilic residue. This pentapeptide forms a very sharp *γ* turn between strand *β*5 and *α*-helix C named the “nucleophilic elbow.” The conformation of this strand-loop-helix motif causes the nucleophilic residue to adopt energetically unfavorable backbone dihedral angles that impose steric constraints on vicinal residues [37, 39, 42]. The “nucleophilic elbow” is the most conserved structural feature of the *αβ* hydrolase fold [38]. In a typical *αβ* hydrolase fold, the catalytic acid is situated in a reverse loop located after strand *β*7, interacting with the catalytic histidine through a hydrogen bond. However, in some enzymes, the catalytic acid can be found after strand *β*6 [37, 42]. Lipases are the only example of *αβ* hydrolases that possess a glutamic acid in the catalytic triad [43]. Histidine is the only residue of the catalytic triad that is absolutely conserved. This residue is located in a loop positioned after strand *β*8. The shape and length of this loop may differ considerably among various members of this family [37, 42].

### 3.2. Enzyme Mechanism

Substrate hydrolysis starts when the oxygen atom of the catalytic serine attacks the carbon atom of the ester linkage carbonyl group, generating a tetrahedral intermediate that makes hydrogen bonds with backbone nitrogen atoms in the “oxyanion hole.” This stabilizes the negatively charged transition state that occurs during hydrolysis. An alcohol is released, leaving the acyl-lipase complex behind, which is ultimately hydrolyzed releasing free fatty acid and regenerating the enzyme [3, 37, 43, 44]. Petersen et al. mapped the electrostatic surface of several lipases and esterases, showing that the active site of these enzymes is negatively charged in their optimal pH range (pH 6–10) [45]. Thus, after the ester cleavage, the ionized carboxylic acid is immediately expelled from the active site due to electrostatic repulsion between its negatively charged carboxyl group and the negative electrostatic potential of the active site, in the so-called “electrostatic catapult” mechanism.

### 3.3. The Lid Domain

Many lipases exhibit a mobile subdomain called lid, which controls the access of substrate molecules to the catalytic center. Crystallographic structures have shown that this domain is able to adopt two distinct conformations: the closed and the opened states. In the closed state, the active site is not accessible to solvent and, as a consequence, enzyme's surface is mainly hydrophilic rendering the lipase inactive. In the opened state, the active site becomes accessible revealing a large hydrophobic surface that makes the enzyme functional [41, 46]. The activation mechanism through which the movement of the lid operates is still poorly understood. Depending on the structural architecture of the lid, several transition mechanisms have been proposed. In lipases with a lid consisting of only one propeller, the transition has been suggested to be a fast rigid body motion. In contrast, in lipases with a more complex lid, such as* Candida rugosa* lipase, the secondary structure of the lid changes during the opening process and, hence, a partial refolding is expected, which can be a bottleneck to the enzyme kinetics [47]. The conformational rearrangement for lid opening is related to the phenomenon of interfacial activation [41]. Some lipases exhibit a significant increase in their enzymatic activity when the substrate concentration exceeds the solubility limit, that is, when the substrate is a hydrophobic interface [39, 48].

Characterization of large conformational transitions at the atomic level in real time through biophysical experimental methods remains a challenge [49]. X-ray techniques provide essential information about the static conformational states but offer a poor dynamic view [41]. NMR spectroscopy is not widely used due to limitations of the technique for high molecular weight proteins, which is the case for many lipases, and only a few lipases as cutinase and* Pseudomonas mendocina* lipase have been structurally characterized by solution NMR [46]. Thus, in an attempt to predict and understand the conformational changes of macromolecular systems, other tools as molecular dynamics simulation have gained considerable attention. A series of molecular dynamics (MD) studies have been performed in order to gain information on conformational changes of the lid domain and the role played by the environment (solvent, pH, and temperature) and by the dynamic properties of various lipases. However, few studies have investigated all aspects of the transitions between open and closed conformations and the molecular details of this important feature of lipase function remain unknown [41].

### 3.4. What Have We Learned from the Three-Dimensional Structures of Lipases?

Through the past few decades, many studies have been conducted on the three-dimensional structure of lipases. X-ray crystallography has been the most used technique to thoroughly describe enzyme's catalytic activity and high selectivity, as well as to reveal the structural basis for interfacial activation.

The first crystal structures of lipases from the fungi* Rhizomucor miehei *[50, 51] and* Geotrichum candidum *[52] were solved in 1990 and 1991, respectively. In 1993, the first crystal structure of a bacterial lipase, from* Pseudomonas glumae* [53], was determined. The structure of this bacterial lipase from the I.2 family revealed for the first time the identity of the catalytic triad and the presence of a lid that controls substrate access to the active site. Although its acidic group was not necessary for lipase function, another aspartate located at position 241 was discovered to be essential for enzyme activity; this residue is capable of binding a calcium ion that is responsible for maintaining protein's stability [53]. Further studies have demonstrated that bacterial lipases from the I.5 family, which also contain a single calcium binding site, exhibit similar characteristics [54].

In 1993, another fungal lipase structure was resolved. The* Candida rugosa* lipase (CRL) structure has allowed a comparison with its homolog from* Geotrichum candidum* and new insights on interfacial activation were unraveled. In contrast to the crystal structure of* Geotrichum candidum* lipase, which has its active site inaccessible from the solvent and covered by loops, CRL has three loops in the vicinity of the active site and its interfacial activation is associated with conformational modifications involving those loops [55]. In 1994, another conformation corresponding to the closed state of this enzyme was crystallized, and only one single loop that occluded the active site in the inactive state was found to be involved in structural rearrangement. In the open form, a large hydrophobic surface area around the active site is exposed and a 9% increase in the total hydrophobic surface of the enzyme is observed [56].

The first crystallographic study of lipase B from* Candida antarctica*, the most widely used fungal lipase in biotechnological processes, was presented in 1994 [57]. CalB contains only seven *β*-strands and thus deviates from the traditional *α*/*β*-hydrolase fold. In addition, the conserved pentapeptide GxSxG located around the catalytic serine is different from most lipases in CalB, with the first glycine being replaced by a threonine residue (TWSQG). The crystal structure showed a quite narrow and deep channel that culminates in an open active site that contains an oxyanion hole. The shape of this channel is probably responsible for the high stereospecificity of the enzyme. A putative lid was also identified based on the mobility of *α*-helix 5 [57]. In contrast, lipase A from* Candida antarctica*, CalA, had its structure solved only fourteen years later revealing a much larger lid composed of 92 residues [58]. Molecular dynamics simulations of CalB in explicit organic solvents (methanol chloroform (CL3), isopentane (ISO), toluene (TOL), and cyclohexane (CHE)) showed a 10% decrease in the hydrophilic and a 1% increase in the hydrophobic surface area of the protein. The dynamics of CalB proved to be largely dependent on the dielectric constant of the solvent, showing high flexibility in water and low flexibility in organic solvents. A significant increase in the number of water molecules bound to the enzyme's surface was observed, decreasing the dielectric constant and spanning the water network [59].

It was only in 1997, seven years after the determination of the first crystal structure of* Rhizomucor miehei* lipase (Rml), that molecular dynamics simulations on free and dialkyl phosphate-bound Rml were performed. This study revealed that hydrophobic residues are exposed while polar residues are buried during the activation process, which is caused by a displacement of the active site helix. Helices showed to be relatively rigid and motions were mostly observed in loop regions, mainly in loops Gly35-Lys50 and Thr57-Asn63 [60]. Further studies using Rml complexed with a substrate (ester) or a product (fatty acid) in the presence of a lipid aggregate have demonstrated that the active site lid opens wider in the presence of a lipid patch consisting of substrate molecules than in an aqueous environment [61].

In the early 2000s, the first crystal structure of a bacterial lipase from the I.1 family was solved.* Pseudomonas aeruginosa* PAO1 lipase showed a variant of the *α*/*β* hydrolase fold without the first two *β*-strands and one *α*-helix (*α*E). A stabilizing intramolecular disulfide bridge is formed between Cys183 and Cys235 due to helix *α*E absence. The active site loop containing the catalytic histidine is stabilized by the coordination of a calcium ion. Three pockets that accommodate the sn-1, sn-2, and sn-3 fatty acid chains were observed. The size of the acyl pocket and its interactions with the substrate, specifically with the sn-2 fatty acid chain, are the predominant determinants of the enzyme's regio- and enantio-preference [62].

In 2001, an example of one of the few lipases that do not exhibit interfacial activation in oil-water interfaces was crystallized. LipA from* Bacillus subtilis* (I.4 family of bacterial lipases) showed a compact minimal *α*/*β*-hydrolase fold with a six-stranded parallel *β*-sheet surrounded by five *α*-helices. No lid domain was observed and the catalytic serine was solvent exposed [63].

Tyndall et al. resolved the first crystallographic structure of a lipase from a thermophilic organism.* Bacillus stearothermophilus* P1 (BSP) lipase shared less than 20% amino acid sequence identity with any other previously crystallized lipase [64]. Its structure contains significant insertions in the canonical *α*/*β* hydrolase fold and a zinc binding site that may be important for thermal stability. In addition, BSP lipase has significantly more salt bridges and *α*-helical content, as well as proline and aromatic residues than any other previously analyzed lipase.

In the past decade, several structural studies have been performed on members of the I.3 lipase family. This unique bacterial lipase family contains two lids covering the active site and is characterized by the presence of three calcium binding sites [54, 65]. The Ca1 site is required for the full opening of the active site by anchoring lid1, the most common lipase lid. Ca2 and Ca3 sites are responsible for stabilizing the enzyme structure, while Ca2 is also required for enzyme full activation. In order to clarify the role of lid2,* Pseudomonas *sp. lipase MIS38 (PML) was used as a model and a lid2 deletion mutant (DL2-PML) was constructed. The crystal structures showed that mutant and native proteins required calcium for lipase activity, suggesting that the enzyme only exhibits activity when lid1 is fully open. The comparison of DL2-PML models in a closed and open conformation with the crystal structures of PML suggests that the hydrophobic surface area provided by lid1 and lid2 in an open conformation is considerably decreased by lid2 deletion. This study proposed that this hydrophobic surface area is necessary to hold firmly the micellar substrates in the active site and, therefore, lid2 is essential for interfacial activation of PML [65]. Previous studies had shown the importance of lid2 for the full activation of PML. Molecular dynamics simulations of PML in the open conformation showed that lid2 closes first, while lid1 stays opened, in the absence of micelles. Similarly, in the absence of Ca^2+^ and in the presence of octane or trilaurin micelles, molecular dynamics simulations of PML in the closed conformation showed that lid1 opens, while lid2 remains closed. These results suggest that Ca1 is necessary not only for fully opened conformation of lid1 but also for the initiation of subsequent opening of lid2 [66].

Recently, the thermoalkalophilic lipase from* Geobacillus zalihae* (T1 lipase from the I.5 family) had its crystal structure in its closed conformation resolved. Based on the structural analysis of lipases from the I.5 family, it was proposed that the lid domain includes helices *α*6 and *α*7 connected by a loop. The activation process is governed by interfacial activation coupled with a temperature-switch activation, as the large structural rearrangement of the lid domain was only observed in the water-octane interface caused by the interaction between the hydrophobic residues of the lid with the octane solvent [67].

Another recent work used a computer-aided software to study the predicted structure and function of the psychrophilic lipase AMS8 (from the psychrophilic* Pseudomonas *sp. obtained in the Antarctic soil). MD simulations were performed at different temperatures for the analysis of structural flexibility and stability. The results showed that the enzyme is most stable at 0°C and 5°C. The N-terminal catalytic domain is more stable than the C-terminal noncatalytic domain, even though the noncatalytic domain displays higher flexibility [68].

Lipases are remarkable biocatalysts used in a variety of bioprocesses. However, in some fields, the industrial application of lipases is still restricted by their high costs. This motivates researches worldwide to find novel strategies to create enzymatic preparations with a more cost-effective profile. Structural information plays an important role in designing lipases for specific purposes. Recent progress in protein engineering and structure-based rational design has led to the customization of lipases for different bioprocesses.

## 4. Tailor-Made Lipases: The Importance of the Structural Knowledge

Traditionally, the use of enzymes for biocatalysis, in particular lipases, began by employing proteins from millions of years of evolution to accelerate chemical reactions, often different from those they have evolved to do. In this “conventional paradigm,” the desired application is frequently different from the natural function of the enzyme. The development of the processes was thus limited by the characteristics of the biocatalyst (i.e., catalytic activity, stability, and enantioselectivity) [69, 70].

Over the past two decades, the advent of techniques such as X-ray crystallography, NMR spectroscopy, and site-specific mutagenesis allowed a better understanding of protein sequence, structure, and function relationships. Because most enzymes from nature do not meet the requirements for a large-scale application, aspects such as chemo-, regio-, and stereoselectivity need to be enhanced in order to establish the process [70–72].

In the “ideal biocatalyst paradigm,” the catalytic characteristics of a target enzyme are often designed by protein engineering, thus building the biocatalyst for a specific optimized process [69]. The paradigm shift brings the focus from the process to the catalyst, which is tailor-made for the desired application.

Protein engineering techniques generally use two different approaches: directed evolution and rational design. The first approach consists in making a series of random mutations in the gene that codes for the target protein, creating a library of transformants that will then be screened for a specific property [70, 73]. There are various techniques to obtain the core steps of directed evolution—mutation, recombination, and screening or selection. The most used are error-prone PCR, which maximize the error rate of DNA polymerase during the PCR reaction [74, 75] and DNA shuffling, which consists in digesting a gene with endonucleases creating a pool of random DNA fragments that can be reassembled into a full-length gene. The DNA fragments anneal with each other based on homology and when fragments from one copy of a gene anneal with those of another copy, recombination occurs causing a template switch [76–78]. The main bottleneck in this approach is the infrastructure necessary to work with a large library of variants (10^3^–10^10^), often requiring high-throughput methods and powerful tools to evaluate the catalysts [73].

The second approach, rational design, preselects promising target sites to make specific changes in the catalyst based on information from protein sequence, structure, and function. This approach may greatly reduce the need for large libraries, focusing on smaller, higher quality ones in order to minimize the screening/selection process [69, 73, 79]. Researchers often look for regions of interest that can be important to the specific target characteristic. Well-conserved function domains, active sites, and key structure points are the focus of the technique. For many enzymes it may be sufficient just to target regions near (10–30 amino acids) the active sites of the proteins in order to obtain significant results in the desired phenotype [73, 80, 81].

However, despite recent advances in this field, engineering an arbitrary protein remains a daunting challenge, because the rules defining the relations among protein sequence, structure, and function are still not entirely understood [82]. To overcome this problem, many hybrid approaches have been used, such as circular permutation, iterative saturation mutagenesis (ISM), combinatorial active-site saturation testing (CASTing), cassette mutagenesis, restricted libraries, and structure-guided consensus [79, 83, 84].

Current literature has many examples of protein engineering using lipases. As multifunctional as these catalysts are, there are examples of directed evolution and rational design towards many characteristics.  Table 1 encompasses successful cases of lipases improvement between 2007 and 2013.

*Candida antarctica* Lipase B (CalB) is a well-known lipase used for many industrial purposes. Its versatility has led to various paths of optimization throughout the past decade. Lutz and Patrick reported several of these changes made over CalB structure in a comprehensive review [85].* Pseudomonas aeruginosa* Lipase A is another well-studied lipase, which had its enantiomeric ratio (E) towards 2-methyldecanoic acid increased from 1.1 to 581 through a series of sequential combined approaches, such as error-prone PCR and ISM [86].

Liebeton et al. used directed evolution to enhance the enantioselectivity of the extracellular lipase from* Pseudomonas aeruginosa* (PAL) [90]. Successive rounds of random mutagenesis by ep-PCR and saturation mutagenesis resulted in increased enantioselectivity from *E* = 1.1 for the wild-type enzyme to *E* = 25.8. This mutant (94G12) showed 5 substitutions (S149G, S155F, V47G, V55G, and S164G) with higher conformational flexibility by accumulation of glycine residues, resulting in modifications of interactions between important structures involved in the catalytic site and oxyanion hole.

Altering optimum pH by structural modifications has been shown by Neves-Petersen et al. using the triglyceride lipase/cutinase from* Fusarium solani pisi *[86]. Previous studies suggested that this enzyme operates through the electrostatic catapult model. Knowing that catalytic Asp175 and Glu44 are key residues involved in this mechanism, mutations E44A (charge removal), E44 K (charge reversal), and T45P were proposed in order to pinpoint the role of these electrostatic changes. Thr45, a neighbouring residue, was mutated with the aim of shifting the spatial location of Glu44. Typically, the substitution of Glu44 pushes the onset of the active site negative potential towards a more alkaline condition, increasing pH optima.

Structural studies and modeling of CalB allowed a great range of experiments to improve its selectivity and thermal stability. Mutation S74A showed a higher enantioselectivity toward 1-chloro-2-octanol. Replacement of a threonine near the active site by valine causes the loss of lipase activity, which is restored when 2-hydroxy-propanoate is used as a substrate. This mutant also presents improved enantioselectivity [40]. Mutants 23G5 (V210I and A281E) and 195F1 (derived from 23G5 with one additional mutation, V221D) showed over a 20-fold increase in half-life at 70°C in comparison to the wild-type CalB, by decreasing mutants propensity to aggregate in the unfolded state. Mutations V221D and A281E are critical for lipase stability, while V210I had only a marginal effect. The mutants catalytic efficiencies against p-nitrophenyl butyrate and 6,8-diuoro-4-methylumbelliferyl octanoate were higher than that for wild-type CalB [91].

NMR analyses of* Pseudomonas mendocina *lipase and its F180P/S205G mutant indicated virtually identical structures with notable differences in local dynamics. These substitutions resulted in a mutant with higher activity and stability for use in washing powders, for instance. While both protein cores are very rigid and widely protected from H/D exchange, specific mutations can stabilize the helices *α*1, *α*4, and *α*5 and destabilize the hydrogen bond network of the *β*-sheet *β*7–*β*9 [92].

Kamal et al. obtained a mutant with twelve stabilizing mutations (A15S, F17S, A20E, N89Y, G111D, L114P, A132D, M134E, M137P, I157 M, S163P, and N166Y) named 6B by performing multiple rounds of directed evolution and mutation by recombination on the lipase A of* Bacillus subtilis *[93]. Eleven of these mutations are involved in better anchoring of loops or increasing their rigidity through substitution of any amino acid by a proline. More importantly, three of the stabilizing mutations (A132D, M134E, and I157 M) are adjacent to two residues of the catalytic triad (D133 and H156), causing increased rigidity of the active site. Interestingly, this increased stiffness resulted in a higher activity. Lipase 6B showed a melting temperature of 22°C and a thermodynamic stability of 3.7 kcal/mol higher than the wild-type protein.

More recently, two mutants (D311E and K344R) of T1 lipase isolated from* Geobacillus zalihae *were constructed to introduce an additional ion pair at the inter- and the intraloop, respectively. The stability of the wild-type and mutant lipases was studied using circular dichroism. The *T*_*m*_ for wild-type lipase and mutants D311E and K344R were approximately 68.52°C, 70.59°C, and 68.54°C, respectively. Analysis of D311E lipase crystal structure revealed an additional ion pair around E311 that may regulate the stability of this mutant at high temperatures [94].

In conclusion, enzyme engineering is a full blossoming field with many interesting works and a myriad of possibilities. Coupling directed evolution with structure-based rational design appears to bring lipase catalysis closer to the “ideal biocatalyst paradigm,” enabling the production of more active, selective, and stable catalysts.

## Figures and Tables

**Table 1:** Examples of lipase improvement using protein engineering (from 2007 to 2013).

Enzyme	Protein engineering approach	Substrate	Optimization	Reference
Lipase B from *Candida antarctica* (CalB)	Circular permutation	2-(3-Fluoro-4-phenyl-phenyl)propionic acid and others	*E*_wt_ (*R*) = 25 *E*_ep_ (*R*) = 40	Qian et al. [87]

*Rhizomucor miehei* lipase	Site-directed mutagenesis	*p*-Nitrophenyl caprylate	5-fold more thermal stability at 60°C	Han et al. [88]

*Bacillus pumilus* lipase	DNA shuffling	*p*-Nitrophenyl palmitate	8-fold specific activity 9-fold half life	Akbulut et al. [78]

*Rhizopus chinensis* lipase	Error-prone PCR/DNA shuffling	*p*-Nitrophenyl palmitate	20°C enhancement in thermal stability	Yu et al. [77]

Lipase A from *Pseudomonas aeruginosa* (PAL)	ISM	2-Methyldecanoic acid p-nitrophenyl ester and other derivatives	*E*_wt_ (*S*) = 1.1 *E*_ep_ (*S*) = 594	Reetz et al. [89]

Lipase from *Yarrowia lipolytica* (Lip2p)	Rational design	2-Bromo-phenylacetic acid ethyl ester and 2-bromo-o-tolylacetic acid ethyl ester	*E*_wt_ (*S*) = 5.5 *E*_ep_ (*S*) = 59 and *E*_wt_ (*S*) = 27 *E*_ep_ (*S*) = 111	Bordes et al. [75]

Lipase A from *Candida antarctica* (CalA)	CASTing	2-Phenyl propanoicacid p-nitrophenylester	*E*_wt_ (*S*) = 20 *E*_ep_ (*R*) = 276	Engström et al. [74]
